# Supplementary material for: Machine Learning-Based Integration of High-Resolution Wildfire Smoke Simulations and Observations for Regional Health Impact Assessment
Source: Int J Environ Res Public Health. 2019 Jun 17;16(12):2137. doi: 10.3390/ijerph16122137 (PMC6617359; doi:10.3390/ijerph16122137)
Supplement: Supplementary file 1 [file ijerph-16-02137-s001.pdf]

Supplementary Materials

Machine Learning-Based Integration of High-Resolution Wildfire Smoke Simulations and Observations for Regional Health Impact Assessment

Yufei Zou [1,*], Susan M. O'Neill [2], Narasimhan K. Larkin [2], Ernesto C. Alvarado [1], Robert Solomon [1], Clifford Mass [3], Yang Liu [4], M. Talat Odman [5], and Huizhong Shen [5]

1 School of Environmental and Forest Sciences, University of Washington, Seattle, WA 98195, USA; alvarado@uw.edu (E.C.A.); robert.airfire@gmail.com (R.S.)
2 Pacific Wildland Fire Sciences Laboratory, U.S. Forest Service, Seattle, WA 98103, USA; smoneill@fs.fed.us (S.M.O.); larkin@fs.fed.us (N.K.L.)
3 Department of Atmospheric Sciences, University of Washington, Seattle, WA 98195, USA; cmass@uw.edu
4 Rollins School of Public Health, Emory University, Atlanta, GA 30322, USA; yang.liu@emory.edu
5 School of Civil and Environmental Engineering, Georgia Institute of Technology, Atlanta, GA 30332, USA; talat.odman@ce.gatech.edu (M.T.O.); shenhz2008@gmail.com (H.S.)
* Correspondence: yzou2017@uw.edu;

Contents of this file:

Equations S1-S4
Figures S1-S5

Figure S1. The statistics of (a) spatial correlation coefficients and (b) RMSE values for all AOD modeling results from 08/15/2017 to 09/14/2017.

Figure S2. The CATS overpass track (the green line) over the WRF-CMAQ surface $PM_{2.5}$ concentration field (color shading; unit: μg m^{-3}) from the SENS experiment at 11:00 UTC (04:00 PDT) on 09/07/2017.

Figure S3. Modeling performance comparison in terms of RMSE with different *mtry* parameter settings in the RF method.

Figure S4. Modeling performance comparison in terms of RMSE with different *shrinkage* and *interaction.depth* parameter settings in the GBM method.

Figure S5. Comparisons of the non-fire CMAQ_CTRL simulated $PM_{2.5}$ surface concentrations with the AirNow ground observations in June-July, 2017, before the fire episode. (a) monthly averaged $PM_{2.5}$ concentrations (unit: μg m^{-3}) based on the AirNow observations; (b) fractional biases (unit: 100%) based on the monthly averaged CMAQ_CTRL simulations and the AirNow observations.

Equations:

The equations of statistical metrics used for the modeling performance evaluation and comparison in the main text are listed below:

Mean absolute error (MAE):

$$MAE = \frac{\sum_i^n abs(\widehat{y_i} - y_i)}{n}, \quad \text{(S1)}$$

Fractional bias (FB):

$$FB = \frac{\sum_i^n (\widehat{y_i} - y_i)}{\sum_i^n y_i} \times 100\%, \quad \text{(S2)}$$

R-squared (R^2):

$$R^2 = \frac{\sum_i^n (\widehat{y_i} - \bar{y})^2}{\sum_i^n (y_i - \bar{y})^2} = 1 - \frac{\sum_i^n (y_i - \widehat{y_i})^2}{\sum_i^n (y_i - \bar{y})^2}, \quad \text{(S3)}$$

Root mean squared error (RMSE):

$$RMSE = \sqrt{\frac{\sum_i^n (\widehat{y_i} - y_i)^2}{n}}, \quad \text{(S4)}$$

In Equations S1-S4, y_i is the ith observed value, $\widehat{y_i}$ is the ith predicted value, $\bar{y}$ is the mean value of all observations, and n is the sample size of observations.

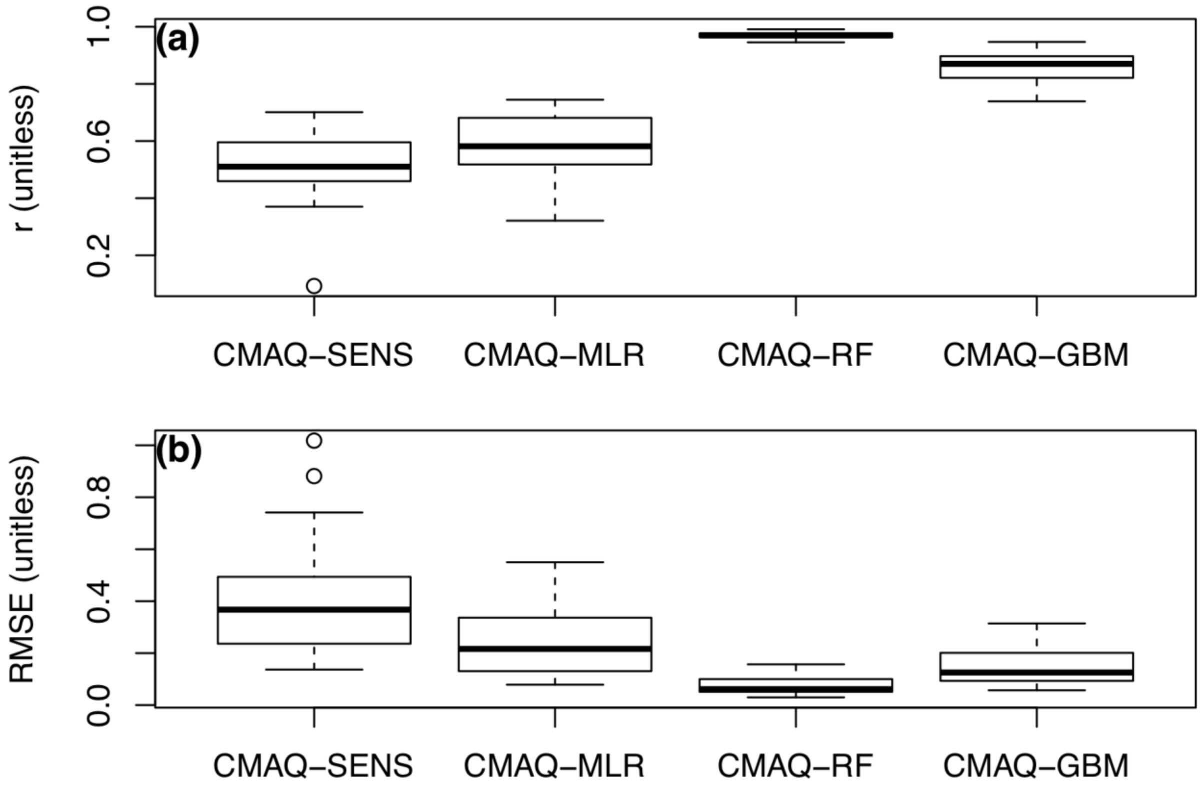

Figure S1. The statistics of (a) spatial correlation coefficients and (b) RMSE values for all AOD modeling results from 08/15/2017 to 09/14/2017.

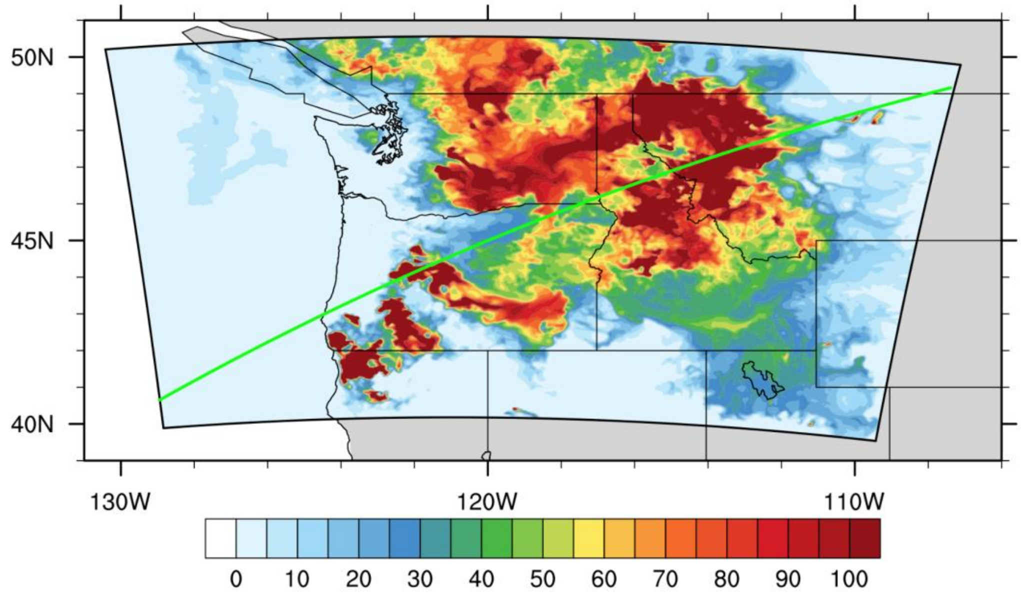

Figure S2. The CATS overpass track (the green line) over the WRF-CMAQ surface $PM_{2.5}$ concentration field (color shading; unit: μg m^{-3}) from the SENS experiment at 11:00 UTC (04:00 PDT) on 09/07/2017.

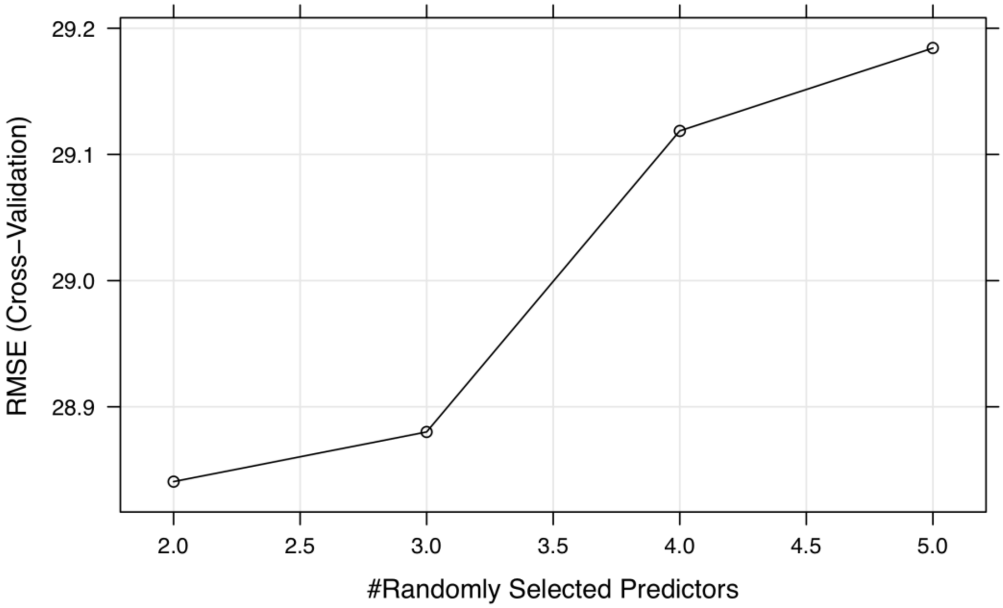

Figure S3. The modeling performance comparison in terms of RMSE with different *mtry* parameter settings in the RF method. Here *mtry* is the number of variables randomly sampled as candidates at each split.

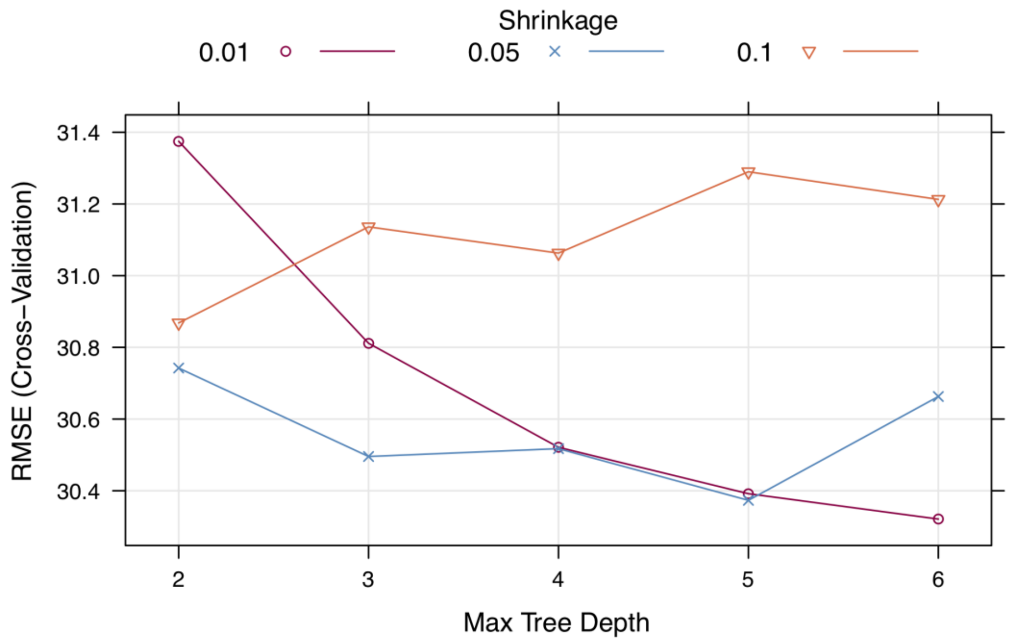

Figure S4. The modeling performance comparison in terms of RMSE with different *shrinkage* and *interaction.depth* parameter settings in the GBM method. Here *shrinkage* is the learning rate applied to each tree in the expansion, and *interaction.depth* is the maximum depth of each tree allowing variable interactions.

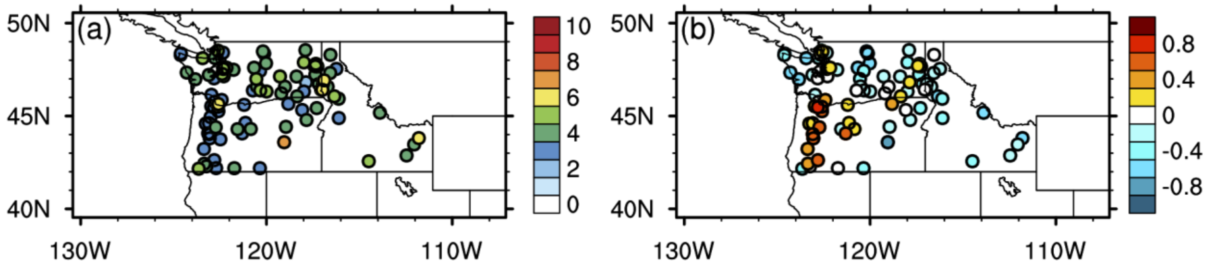

Figure S5. Comparisons of the non-fire CMAQ_CTRL simulated $PM_{2.5}$ surface concentrations with the AirNow ground observations in June-July, 2017, before the fire episode. (a) monthly averaged $PM_{2.5}$ concentrations (unit: μg m^{-3}) based on the AirNow observations; (b) fractional biases (unit: 100%) based on the monthly averaged CMAQ_CTRL simulations and the AirNow observations;
